# Novel Strategies for Improved Treatment of O6-Methylguanine-DNA Methyltransferase Promoter-Methylated Glioma

**DOI:** 10.18103/mra.v13i9.6944

**Published:** 2025-09-25

**Authors:** Juan C. Vasquez, Ranjit S. Bindra, Susan E. Gueble

**Affiliations:** 1 Department of Pediatrics, Yale School of Medicine, New Haven, CT; 2 Department of Therapeutic Radiology, Yale School of Medicine, New Haven, CT; 3 Department of Pathology, Yale School of Medicine, New Haven, CT

## Abstract

Adult diffuse gliomas are primary brain tumors notorious for leading to devastating neurologic consequences from both tumor progression and therapeutic interventions. The arsenal of current established treatments primarily includes surgery, radiotherapy, and DNA alkylating chemotherapy agents. Unfortunately, even with aggressive treatments, long-term cure is typically not attainable, except in certain cases of low-grade gliomas amenable to complete surgical resection. Grade 4 glioblastoma (GBM) represents the most aggressive and most common type of glioma in adults, is often resistant to current therapies, and is associated with a median survival of approximately 15 months. While biomarker-based therapies for gliomas are limited, O6-methylguanine-DNA methyltransferase (MGMT) is one well-established prognostic marker in GBM and is associated with improved response to the alkylating agent temozolomide (TMZ). Methylation of the MGMT promoter leading to loss of MGMT expression occurs in approximately half of GBMs and 70–80% of anaplastic and low-grade gliomas. While MGMT promoter-methylated gliomas are responsive to TMZ, a characteristic resistance mechanism of mismatch repair loss often emerges, resulting in recurrent drug-resistant disease. In prior work, we identified a new TMZ derivative “KL-50” which overcomes resistance to TMZ driven by loss of mismatch repair in preclinical glioma models. KL-50 functions via a novel DNA-modifying mechanism involving evolution of a primary alkyl lesion to a DNA interstrand crosslink specifically in the absence of MGMT. Research is ongoing to establish this new class of agents as a potential improved therapy in human gliomas. In this review, we provide an overview of the history and evolution of alkylator use in GBM, discuss the mechanisms and pitfalls of current therapies including toxicity or susceptibility to resistance mechanisms, and present the potential of a new wave of DNA modifiers to improve outcomes in gliomas.

## I. Introduction

Glioblastoma (GBM), IDH–wild-type, is the most common and aggressive form of diffuse glioma and accounts for the majority of malignant primary brain tumors in adults. It is defined by its highly infiltrative growth, rapid progression, and poor prognosis, with a median survival of approximately 15 months and a five-year survival rate of less than 10% despite maximal therapy^1,2^. The current standard-of-care, which includes surgical resection followed by radiation and concomitant and adjuvant temozolomide (TMZ), offers only modest, transient benefit^3,4^. Other diffuse gliomas, including IDH-mutant astrocytomas and oligodendrogliomas, follow a more protracted clinical course but remain incurable and ultimately progress to treatment-refractory disease. Even among patients with WHO grade 2–3 diffuse gliomas, median overall survival ranges from 5 to 10 years, with outcomes highly dependent on age, extent of resection, and molecular subtype^5^. Across this spectrum, recurrence is common and effective salvage therapies are lacking^6^.

The primary alkylating agent used in the treatment of diffuse gliomas, TMZ, is limited by predictable resistance mechanisms. In tumors with intact O6-methylguanine DNA methyltransferase (MGMT) expression, TMZ-induced O6-methylguanine lesions are rapidly repaired, preventing cytotoxicity^7–9^. In MGMT-silenced tumors, which are typically more responsive to TMZ, selective treatment pressure frequently leads to acquired mismatch repair (MMR) deficiency, most often through mutations in MSH6^9–13^. These recurrences can exhibit a hypermutator phenotype yet remain unresponsive to alkylator rechallenge and immune checkpoint blockade^13–15^. Nitrosoureas, such as BCNU (carmustine) and CCNU (lomustine) provide only incremental benefit and are constrained by significant hematologic toxicity and lack of tumor specificity^16^. The need for therapies that can overcome TMZ resistance while maintaining a favorable therapeutic index remains unmet in both GBM and other molecularly defined diffuse gliomas.

In this review, we trace the evolution of alkylating agents in diffuse glioma, examine the molecular determinants of resistance to current treatments, and explore the therapeutic potential of novel agents engineered to address these shortcomings. We focus on KL-50, a next-generation imidazotetrazine designed to induce MGMT-dependent, MMR-independent DNA crosslinks, representing a rational approach to selectively target MGMT-silenced, treatment-refractory gliomas.

## II. Early history and evolution of alkylator use in glioblastoma

### NITROSOUREAS: BIFUNCTIONAL ALKYLATORS

Alkylating agents have played a central role in the treatment of GBM for over five decades. The introduction of nitrosoureas in the 1970s marked the first class of chemotherapeutics with demonstrable activity against malignant gliomas. Carmustine and lomustine (also referred to as BCNU and CCNU, respectively) were among the first chemotherapeutics to penetrate the blood–brain barrier and produce responses in patients with malignant gliomas^17,18^. These agents act as bifunctional alkylators, inducing DNA damage by covalently modifying the O6 position of guanine and generating interstrand crosslinks, which block DNA replication and transcription and ultimately lead to cell death^7,8^.

A landmark randomized trial conducted by Walker et al. demonstrated that adding carmustine to postoperative radiotherapy showed modest prolongation of survival, establishing chemotherapy as a viable adjunct to radiation in high-grade glioma^17^. Despite this modest benefit, nitrosoureas were associated with significant hematologic toxicity. Subsequent studies from cooperative groups evaluated various combinations and schedules of nitrosoureas, such as PCV (procarbazine, lomustine, and vincristine), with survival outcomes ranging from 9 to 12 months and 1-year survival rates rarely exceeding 30%^19,20^. Toxicity remained a persistent issue, often limiting dose intensity and duration of therapy.

Despite these drawbacks, nitrosoureas continue to play a role in the recurrent GBM setting, particularly lomustine due to its oral formulation. For example, the BELOB trial, a randomized phase II study, compared lomustine alone, bevacizumab alone, or the combination in recurrent GBM. The combination arm showed improved 9-month overall survival (63% vs. 38% for lomustine alone), although the trial was not powered for definitive comparisons^21^. This study laid the groundwork for the EORTC 26101 phase III trial, which confirmed a modest improvement in progression-free survival with the addition of bevacizumab to lomustine (4.2 vs. 1.5 months) but no difference in overall survival (9.1 vs. 8.6 months). Notably, roughly 50% of patients experienced grade ≥3 hematologic toxicity^22^. These findings underscore both the historical importance and therapeutic limitations of nitrosoureas in GBM.

### TEMOZOLOMIDE: A MONOFUNCTIONAL ALKYLATOR

Seeking improved efficacy and tolerability, researchers developed temozolomide (8-carbamoyl-3-methylimidazo[5,1-d]-1,2,3,5-tetrazin-4(3H)-one), a second-generation imidazotetrazine compound originally described in 1987 and currently the most widely used alkylating agent for treatment of GBM and lower grade diffuse gliomas^7,8,23^. In contrast to the bifunctional alkylators described previously which produce DNA crosslinking, TMZ is a monofunctional methylating agent. TMZ, a prodrug, hydrolyzes under physiological conditions to the linear triazene compound 5-(3-methyltriazen-1-yl)imidazole-4-carboxamide (MTIC), which in turn yields a methyldiazonium cation, the active DNA methylating species^7,23^. DNA methylation preferentially occurs at the N7, N3, and O6 atoms of guanine and N3 atom of adenine, due to the high nucleophilicity of these sites. With the exception of O6-methylguanine, these lesions are substrates for base excision repair enzymes and are readily repaired in most cells. Conversely, O6-methylguanine is a substrate for the direct reversal DNA repair enzyme MGMT, and repair of this lesion depends critically on the cellular activity of MGMT^7,8,24^. Following transfer of the methyl group to a cysteine residue in its active site, MGMT undergoes ubiquitination and subsequent proteasomal degradation. In the absence or depletion of MGMT, persistent O6-methylguanine lesions mispair with thymidine during replication, leading to recognition and processing by the MutSα and MutSβ MMR complexes. Excision of thymidine followed by resynthesis of the DNA mispair ultimately generates a process of “futile cycling” which leads to formation of DNA double-strand breaks and eventual cell death^7,25^.

Temozolomide displayed promising efficacy in mouse tumors and was advanced to single agent trials where it demonstrated activity in recurrent GBM^23,26^. Whereas the first generation imidazotetrazine compound mitozolomide and other bifunctional alkylators discussed above were severely hindered by dose-limiting toxicities including myelosuppression in the clinical setting, TMZ was better tolerated^20,26,27^. In addition, TMZ possesses favorable pharmacokinetic properties with oral bioavailability and penetration of the blood-brain barrier^28,29^. In 2005, the seminal randomized clinical trial reported by Stupp et al. demonstrated a survival benefit with the addition of concurrent and adjuvant TMZ to standard radiotherapy for newly diagnosed GBM^3^.

Importantly, translational studies from this trial identified MGMT promoter methylation as a critical biomarker predicting clinical benefit from TMZ^4^. The MGMT gene promoter contains a 5’ CpG island of ~770 base pairs and 97 CpG sites, beginning ~480 base pairs upstream of the transcription start site and traversing through the first non-coding exon^30^. Cytosine methylation in the MGMT promoter leads to chromatin condensation and exclusion of transcription factor binding, resulting in reduced MGMT expression^31,32^. Hegi et al. found that 45% of GBMs harbored MGMT promoter methylation. Among these patients, median survival increased from 15.3 to 21.7 months with the addition of TMZ, whereas in patients without MGMT promoter methylation, overall survival was not significantly affected^4^. Additional studies have since confirmed MGMT silencing in ~40–50% of GBM and have corroborated the predictive value of silencing with respect to TMZ response^33,34^. In current clinical practice, TMZ is utilized for the upfront treatment of newly diagnosed GBM in combination with external beam radiotherapy following maximal safe surgical resection. While studies suggest a greater benefit to TMZ in MGMT-silenced tumors, the lack of other effective treatment options in MGMT unmethylated tumors has led to its recommended use independent of MGMT status^35,36^. For patients over 70 years of age, TMZ treatment alone can also be considered, particularly for those with MGMT promoter methylated tumors or with a poor performance status^36,37^. Unfortunately, GBM nearly inevitably recurs, and no effective standard-of-care has been established for recurrent or progressive cases.

In lower grade gliomas, MGMT promoter silencing is found at even higher rates, approaching 80% of cases. In this setting, MGMT promoter silencing is strongly associated with mutations in isocitrate dehydrogenase 1 and 2 (IDH-1 and -2)^38,39^. Tumor associated IDH1/2 mutations generate neomorphic gain-of-function status leading to overproduction of the oncometabolite 2-hydroxyglutarate, which acts as a competitive inhibitor of alpha-ketoglutarate-dependent dioxygenases, several of which are implicated in DNA and histone methylation^40–42^. As a result, IDH1/2-mutant gliomas exhibit a CpG-island methylator phenotype (G-CIMP) associated with widespread gene silencing, including MGMT promoter silencing in over 90% of cases^38,39,43,44^.

For WHO grade 3 and high-risk WHO grade 2 astrocytomas, adjuvant TMZ following radiotherapy is typically recommended, stemming from studies demonstrating improved outcomes compared to radiotherapy alone, particularly in IDH-mutant tumors^45,46^. However, while progression-free survival is longer for IDH mutant grade 2–3 gliomas, these tumors still often recur with characteristic signs of TMZ resistance, as discussed further below^15^. The use of TMZ in place of the alternative alkylating regimen PCV (procarbazine, lomustine, vincristine) for oligodendrogliomas defined by 1p/19q codeletion is not yet established, as the phase III CODEL trial is ongoing; however, TMZ remains a commonly used clinical option due to its more favorable tolerability profile^47^. Thus, additional therapies improving upon TMZ for both GBM and lower grade diffuse gliomas are a high clinical priority.

## IV. Mismatch repair: A key resistance mechanism to temozolomide

As described above, in the absence of MGMT, TMZ induced O6-methylguanine lesions mispair with thymidine during replication, leading to recognition and processing by the MutSα and MutSβ complexes. This MMR activity leads to excision of thymidine followed by resynthesis of the DNA mispair, ultimately generating a process of “futile cycling” which causes replication fork collapse, DNA double-strand breaks, and cell death via apoptosis or mitotic catastrophe^7,25,48^.

Loss of MMR function is now recognized as a key mechanism of acquired TMZ resistance in gliomas, particularly in tumors with MGMT promoter methylation. In a seminal study by Hunter et al., targeted sequencing identified somatic inactivating mutations in MSH6 in recurrent gliomas that had been treated with TMZ^49^. Subsequent studies using whole-exome sequencing (WES) and immunohistochemistry (IHC) have corroborated these findings, demonstrating that up to 25–30% of recurrent MGMT-methylated gliomas acquire mutations in core MMR genes, most commonly MSH6, but also MSH2, MLH1, and PMS2^10,13,50–53^. Importantly, MMR loss is shown to be restricted to post-treatment recurrences while absent in untreated primary tumors, strongly implicating acquired MMR deficiency as a major driver of TMZ resistance. This phenomenon is not limited to IDH–wild-type GBM. TMZ-induced hypermutation has been observed in nearly 60% of low-grade, IDH-mutant gliomas that recur with anaplastic transformation following treatment^15,53^.

Mismatch repair deficiency is also relevant in the pediatric setting, where a subset of high-grade gliomas arises in the context of germline mutations in MMR, such as constitutional mismatch repair deficiency (CMMRD)^9,54,55^. These tumors are typically resistant to TMZ at diagnosis due to a pre-existing lack of functional MMR. Although the co-occurring frequency of MGMT-silencing in the setting of germline MMR deficiency, and the role of MGMT promoter methylation in modulating TMZ sensitivity in pediatric patients is unclear, recent studies suggest that MGMT promoter methylation is common in certain molecular subtypes such as H3.3-G34R and H3K27M wild-type tumors^56–59^.

Functionally, MMR deficiency results in tolerance of O6-meG:T mismatches, allowing tumor cells to escape the cytotoxic effects of TMZ. This enables continued proliferation despite ongoing DNA damage and results in the accumulation of somatic mutations, leading to a hypermutator phenotype characterized by elevated tumor mutation burden (TMB) and extensive genomic instability^13,15,60^. Although microsatellite instability (MSI) is a hallmark of MMR deficiency in other cancers, such as colorectal and those arising in the setting of germline MMR deficiency, it is often absent or minimal in gliomas^13,61–64^. This discrepancy likely reflects the subclonal nature and later emergence of TMZ-induced MMR mutations, which provide limited time for MSI to develop, as well as tissue-specific differences in repair pathway activity^13,65,66^. Thus, WES and IHC remain the most reliable methods for detecting MMR deficiency in glioma.

Notably, while a hypermutator phenotype has been associated with improved responses to immune checkpoint blockade (ICB) in other solid tumors, such as melanoma and colorectal cancer, this has not translated to glioma^13,61,62,67^. Recurrent hypermutant, MMR-deficient gliomas exhibit poor responses to ICB, likely due to the poor fitness and subclonal nature of therapy-induced neoantigens and a highly immunosuppressive tumor microenvironment with impaired antigen presentation^13,66^.

Together, these findings underscore that MMR deficiency is a frequent, treatment-emergent mechanism of resistance to TMZ in MGMT-methylated and IDH-mutant gliomas. This phenomenon contributes to the development of a hypermutant, genomically unstable tumor state that resists further alkylator therapy and typically fails to respond to ICB, reinforcing the need for novel therapeutic approaches.

## V. Beyond temozolomide, testing the next wave of DNA modifiers

### BYPASSING O6-METHYLGUANINE-DNA METHYLTRANSFERASE-MEDIATED RESISTANCE

To improve alkylator therapies for GBM and glioma, some efforts have focused on overcoming the intrinsic resistance to TMZ seen in MGMT unmethylated tumors, namely MGMT expression. One approach is the use of O6-methylguanine mimetics, such as O6-benzylguanine (O6BG) or O6-(4-bromothenyl)guanine (lomeguatrib), as MGMT inhibitors. These compounds inactivate MGMT, lead to its cellular depletion, potentiate the activity of TMZ in preclinical models, and have been shown in human studies to deplete MGMT in peripheral blood mononuclear cells and tumors^68–72^. However, combination therapy of these inhibitors with TMZ in human clinical trials has necessitated TMZ dose-reductions because of dose-limiting myelosuppression^71,72^. At these reduced doses, O6BG with TMZ showed limited effectiveness in recurrent GBM or anaplastic gliomas, with objective response rates of 3% and 16% respectively^73^. Lomeguatrib with TMZ has primarily been tested in non-glioma solid tumors, including melanoma and colorectal cancer, and has similarly failed to demonstrate significant response rates despite significant hematologic toxicities^74,75^. Several groups have reported drug delivery approaches to reducing systemic toxicity and enhancing CNS exposure to combined O6BG and TMZ, including PLGA nanoparticles, PEGylated liposomes, and exosomes engineered with GBM cell targeted molecules, but these remain in early preclinical stages of investigation^76–78^.

A second approach has been the derivatization of TMZ to effect deposition of O6-guanyl adducts that are irreparable by MGMT. Various substitutions at the N3-methyl position of TMZ have yielded compounds with cytotoxic activity irrespective of MGMT or MMR expression^79–83^. Modifications at the C8 carboxamide position of TMZ have also been shown to reduce MGMT-mediated drug resistance and improve drug metabolism and pharmacokinetic properties^82–85^.

While these various strategies of MGMT inhibitors or MGMT-independent alkylation broaden the range of tumors potentially amenable to treatment to include the MGMT unmethylated cohort, they also abrogate the potential of MGMT methylation to yield tumor-specific sensitization relative to healthy MGMT-expressing cells. Therefore, as noted, many of these strategies have been shown to be limited by off-target normal tissue toxicity.

### A NEW CLASS OF TUMOR-SELECTIVE DNA MODIFIERS

Using a different strategy, our group sought to develop an improved therapy that would maintain the selectivity for MGMT silenced gliomas, but overcome resistance driven by loss of MMR^86^. While this strategy was not designed to target MGMT unmethylated tumors, it was focused on exploiting the genetic vulnerability in MGMT silenced tumors, thereby establishing a therapeutic index in tumors relative to normal tissues. To this end, a panel of TMZ derivatives was synthesized and screened across glioma models with isogenic expression of MGMT and/or MMR, searching for a compound that would selectively kill MGMT negative cells regardless of MMR status. In this manner, KL-50, a novel 2-fluoroethyl derivative of temozolomide, was identified with the desired MGMT-selective, but MMR-independent activity. In isogenic cell line models, KL-50 displayed approximately 24-fold greater potency in MGMT-negative compared to MGMT-positive cells yet showed no decrease in potency upon loss of MMR. In comparison, loss of MMR led to an approximately 100-fold decrease in TMZ activity. The activity of KL-50 was maintained in cell-derived xenograft glioma models in which it displayed effective tumor control in MGMT-silenced tumors with loss of MMR that were impervious to TMZ. KL-50 was well-tolerated in mice based on mouse bodyweights and blood counts at doses effective for tumor control^86^.

As with TMZ, KL-50 is posited to undergo hydrolysis under physiological conditions, forming a 2-fluoroethyldiazonium ion which deposits 2-fluorethyl lesions on DNA bases. These 2-fluoroethyl DNA lesions are relatively stable given the poor leaving group ability of fluorine (in comparison, for example, to the chloroethyl lesions derived from the bifunctional alkylating agents lomustine or mitozolomide). However, at O6-(2-fluoroethyl)guanine lesions, the fluoride can undergo unimolecular displacement to form an N1,O^6^-ethanoguanine intermediate, which can subsequently be ring-opened by the opposing cytidine residue, generating a DNA interstrand crosslink (ICL). Notably, O6-(2-fluoroethyl)guanosine transforms to N1,O^6^-ethanoguanosine with a half-life of 18.5 hours under physiological conditions^87^, much more slowly than the expected rate of lesion repair, which for O6-ethylguanine is ~3 hours as measured in rat liver DNA^88^. In comparison, the half-life of O^2^-(2-chloroethyl)guanosine, formed by other bifunctional crosslinking agents such as lomustine or mitozolomide, is only 18 minutes, exceeding the rate of MGMT-mediated reversal^89^.

These kinetic considerations suggest a unique ability of KL-50 to form DNA ICLs only in the absence of MGMT, whereas chloroethylating agents form ICLs more indiscriminately. Indeed, in rigorous biochemical studies, O6-(2-fluoroethyl)guanine-containing oligonucleotides slowly produced DNA ICLs (half-life = 80 hours) that were nearly eliminated with the prior addition of purified MGMT^90^. In contrast, O6-(2-chloroethyl)guanine-containing oligonucleotides produced DNA ICLs >10-fold more rapidly (half-life = 6.3 hours) and were only partially reduced with the prior addition of purified MGMT and replaced instead with MGMT-DNA protein crosslinks, which are also a known cause of cell toxicity. In cellulo, KL-50 exposure similarly led to time-dependent formation of ICLs in genomic DNA and delayed induction of markers of DNA damage and replication stress specifically in MGMT-deficient cells^86^. Critically, the formation of DNA ICLs bypasses the need for MMR-induced “futile cycling” to drive toxicity, accounting for the comparable KL-50 activity in both MMR proficient and deficient settings.

KL-50 was also recently investigated in patient-derived models of IDH1/2 wild-type GBM^14^. In two different TMZ-naïve intracranial GBM patient-derived xenograft models, one displaying partial MGMT promoter methylation and the other displaying full MGMT promoter methylation, KL-50 displayed robust monotherapy activity compared to vehicle control, approximately doubling mouse median survival. Furthermore, treatment of one of these models with repeated cycles of TMZ led to loss of expression of multiple MMR proteins, including MSH2, MSH6, MLH1, and PMS2. Upon rechallenge of these post-TMZ, MMR-deficient tumors, KL-50 proved more effective than TMZ, extending median survival from 108 to 140 days (p = 0.02). Finally, consistent with prior studies in established glioma cell lines, knockout of MSH6 in primary GBM cultures induced resistance to TMZ, but not KL-50. Altogether, these studies support the potential of 2-fluoroethylating agents both as upfront therapy for MGMT promoter methylated GBM or in the recurrent post-TMZ setting.

## VI. Conclusions

The development of fluoroethylating agents, such as KL-50, represents a rational approach to overcoming key resistance mechanisms in GBM. Preclinical studies have demonstrated that KL-50 induces DNA interstrand crosslinks selectively in MGMT-silenced tumor models and retains activity in the absence of functional MMR, highlighting its potential to fill a critical therapeutic gap in recurrent, treatment-refractory tumors. In addition to its relevance in MGMT-silenced GBM, KL-50 may have therapeutic utility in IDH-mutant gliomas, which frequently exhibit MGMT promoter methylation and are prone to acquiring TMZ-induced MMR deficiency ^15,38,91^. Moreover, IDH-mutant gliomas harbor broad defects in DNA repair pathways, including homologous recombination and PARP-dependent base excision repair, due to oncometabolite-induced epigenetic dysregulation, potentially rendering them more sensitive to crosslinking agents like KL-50^92–94^. KL-50 also warrants evaluation in pediatric high-grade gliomas, where MGMT silencing and TMZ responsiveness have historically been less well-defined, but the frequency of underlying germline MMR deficiency syndromes is increasingly recognized^9,54,55^. Together, these observations support KL-50’s potential applicability across adult and pediatric gliomas with defined defects in DNA repair.

Future studies should also explore KL-50 in rational combination regimens. Given its unique mechanism of inducing time-dependent DNA interstrand crosslinks in MGMT-silenced cells, KL-50 triggers replication stress and activates downstream DNA repair pathways, including homologous recombination and ATR/CHK1-mediated checkpoint signaling^86^. These responses suggest that there is great potential for combinations with inhibitors of the DNA damage response, such as PARP or ATR inhibitors, to induce synthetic lethality. Furthermore, recent clinical data from the NOA-9 phase III trial also support the rationale for combination alkylator strategies, demonstrating a survival benefit with the addition of lomustine to TMZ in newly diagnosed MGMT-silenced GBM, suggesting that combinations leading to both alkylation damage and DNA crosslinking may enhance antitumor efficacy^95^. The ongoing NRG BN011 trial aims to build on this finding by incorporating more robust stratification and modern trial infrastructure, with the goal of getting FDA-level confirmatory evidence^96^. These findings collectively support the potential for combining TMZ and KL-50, which may enhance the therapeutic index compared to TMZ and lomustine owing to the greater MGMT dependency of KL-50.

Finally, expanding the scope of KL-50 to include extracranial malignancies with MGMT silencing, such as colorectal cancer and other oncometabolite-driven tumors, may further broaden the clinical application of this novel class of agents. Defining predictive biomarkers and resistance mechanisms will be key to optimizing clinical development and realizing the full potential of KL-50.

## References

[1] Ostrom QT, Price M, Neff C, CBTRUS Statistical Report: Primary Brain and Other Central Nervous System Tumors Diagnosed in the United States in 2015–2019. Neuro Oncol. Oct 5 2022;24(Suppl 5):v1–v95. doi:10.1093/neuonc/noac202 36196752 PMC9533228

[2] Poon MTC, Sudlow CLM, Figueroa JD, Brennan PM. Longer-term (>/= 2 years) survival in patients with glioblastoma in population-based studies pre- and post-2005: a systematic review and meta-analysis. Sci Rep. Jul 15 2020;10(1):11622. doi:10.1038/s41598-020-68011-4 32669604 PMC7363854

[3] Stupp R, Mason WP, van den Bent MJ, Radiotherapy plus concomitant and adjuvant temozolomide for glioblastoma. N Engl J Med. Mar 10 2005;352(10):987–96. doi:10.1056/NEJMoa043330 15758009

[4] Hegi ME, Diserens AC, Gorlia T, MGMT gene silencing and benefit from temozolomide in glioblastoma. N Engl J Med. Mar 10 2005;352(10):997–1003. doi:10.1056/NEJMoa043331 15758010

[5] Mair MJ, Geurts M, van den Bent MJ, Berghoff AS. A basic review on systemic treatment options in WHO grade II-III gliomas. Cancer Treat Rev. Jan 2021;92:102124. doi:10.1016/j.ctrv.2020.102124 33227622

[6] Weller M, van den Bent M, Tonn JC, European Association for Neuro-Oncology (EANO) guideline on the diagnosis and treatment of adult astrocytic and oligodendroglial gliomas. Lancet Oncol. Jun 2017;18(6):e315–e329. doi:10.1016/S1470-2045(17)30194-8 28483413

[7] Strobel H, Baisch T, Fitzel R, Temozolomide and Other Alkylating Agents in Glioblastoma Therapy. Biomedicines. Sep 9 2019;7(3)doi:10.3390/biomedicines7030069 PMC6783999 31505812

[8] Temozolomide Kaina B., Procarbazine and Nitrosoureas in the Therapy of Malignant Gliomas: Update of Mechanisms, Drug Resistance and Therapeutic Implications. J Clin Med. Nov 30 2023;12(23)doi:10.3390/jcm12237442 PMC10707404 38068493

[9] Leelatian N, Hong CS, Bindra RS. The Role of Mismatch Repair in Glioblastoma Multiforme Treatment Response and Resistance. Neurosurg Clin N Am. Apr 2021;32(2):171–180. doi:10.1016/j.nec.2020.12.009 33781500

[10] Cahill DP, Levine KK, Betensky RA, Loss of the mismatch repair protein MSH6 in human glioblastomas is associated with tumor progression during temozolomide treatment. Clin Cancer Res. Apr 1 2007;13(7):2038–45. doi:10.1158/1078-0432.CCR-06-2149 17404084 PMC2873832

[11] Shinsato Y, Furukawa T, Yunoue S, Reduction of MLH1 and PMS2 confers temozolomide resistance and is associated with recurrence of glioblastoma. Oncotarget. Dec 2013;4(12):2261–70. doi:10.18632/oncotarget.1302 24259277 PMC3926825

[12] Yip S, Miao J, Cahill DP, MSH6 mutations arise in glioblastomas during temozolomide therapy and mediate temozolomide resistance. Clin Cancer Res. Jul 15 2009;15(14):4622–9. doi:10.1158/1078-0432.CCR-08-3012 19584161 PMC2737355

[13] Touat M, Li YY, Boynton AN, Mechanisms and therapeutic implications of hypermutation in gliomas. Nature. Apr 2020;580(7804):517–523. doi:10.1038/s41586-020-2209-9 32322066 PMC8235024

[14] McCord M, Sears T, Wang W, The novel DNA cross-linking agent KL-50 is active against patient-derived models of new and recurrent post-temozolomide mismatch repair-deficient glioblastoma. Neuro Oncol. Mar 7 2025;27(3):644–651. doi:10.1093/neuonc/noae257 39658092 PMC11889708

[15] Yu Y, Villanueva-Meyer J, Grimmer MR, Temozolomide-induced hypermutation is associated with distant recurrence and reduced survival after high-grade transformation of low-grade IDH-mutant gliomas. Neuro Oncol. Nov 2 2021;23(11):1872–1884. doi:10.1093/neuonc/noab081 33823014 PMC8563321

[16] Fazzari FGT, Rose F, Pauls M, The current landscape of systemic therapy for recurrent glioblastoma: A systematic review of randomized-controlled trials. Crit Rev Oncol Hematol. Jan 2022;169:103540. doi:10.1016/j.critrevonc.2021.103540 34808376

[17] Walker MD, Green SB, Byar DP, Randomized comparisons of radiotherapy and nitrosoureas for the treatment of malignant glioma after surgery. N Engl J Med. Dec 4 1980;303(23):1323–9. doi:10.1056/NEJM198012043032303 7001230

[18] Weller M, Le Rhun E. How did lomustine become standard of care in recurrent glioblastoma? Cancer Treat Rev. Jul 2020;87:102029. doi:10.1016/j.ctrv.2020.102029 32408220

[19] Medical Research Council Brain Tumor Working P. Randomized trial of procarbazine, lomustine, and vincristine in the adjuvant treatment of high-grade astrocytoma: a Medical Research Council trial. J Clin Oncol. Jan 15 2001;19(2):509–18. doi:10.1200/JCO.2001.19.2.509 11208845

[20] Brada M, Stenning S, Gabe R, Temozolomide versus procarbazine, lomustine, and vincristine in recurrent high-grade glioma. J Clin Oncol. Oct 20 2010;28(30):4601–8. doi:10.1200/JCO.2009.27.1932 20855843

[21] Taal W, Oosterkamp HM, Walenkamp AM, Single-agent bevacizumab or lomustine versus a combination of bevacizumab plus lomustine in patients with recurrent glioblastoma (BELOB trial): a randomised controlled phase 2 trial. Lancet Oncol. Aug 2014;15(9):943–53. doi:10.1016/S1470-2045(14)70314-6 25035291

[22] Wick W, Gorlia T, Bendszus M, Lomustine and Bevacizumab in Progressive Glioblastoma. N Engl J Med. Nov 16 2017;377(20):1954–1963. doi:10.1056/NEJMoa1707358 29141164

[23] Stevens MF, Hickman JA, Langdon SP, Antitumor activity and pharmacokinetics in mice of 8-carbamoyl-3-methyl-imidazo[5,1-d]-1,2,3,5-tetrazin-4(3H)-one (CCRG 81045; M & B 39831), a novel drug with potential as an alternative to dacarbazine. Cancer Res. Nov 15 1987;47(22):5846–52. 3664486

[24] Fu D, Calvo JA, Samson LD. Balancing repair and tolerance of DNA damage caused by alkylating agents. Nat Rev Cancer. Jan 12 2012;12(2):104–20. doi:10.1038/nrc3185 22237395 PMC3586545

[25] Roos WP, Batista LF, Naumann SC, Apoptosis in malignant glioma cells triggered by the temozolomide-induced DNA lesion O6-methylguanine. Oncogene. Jan 11 2007;26(2):186–97. doi:10.1038/sj.onc.1209785 16819506

[26] Yung WK, Albright RE, Olson J, A phase II study of temozolomide vs. procarbazine in patients with glioblastoma multiforme at first relapse. Br J Cancer. Sep 2000;83(5):588–93. doi:10.1054/bjoc.2000.1316 10944597 PMC2363506

[27] O’Reilly SM, Newlands ES, Glaser MG, Temozolomide: a new oral cytotoxic chemotherapeutic agent with promising activity against primary brain tumours. Eur J Cancer. 1993;29A(7):940–2. doi:10.1016/s0959-8049(05)80198-4 8499146

[28] Ostermann S, Csajka C, Buclin T, Plasma and cerebrospinal fluid population pharmacokinetics of temozolomide in malignant glioma patients. Clin Cancer Res. Jun 1 2004;10(11):3728–36. doi:10.1158/1078-0432.CCR-03-0807 15173079

[29] Patel M, McCully C, Godwin K, Balis FM. Plasma and cerebrospinal fluid pharmacokinetics of intravenous temozolomide in non-human primates. J Neurooncol. Feb 2003;61(3):203–7. doi:10.1023/a:1022592913323 12675312

[30] Everhard S, Tost J, El Abdalaoui H, Identification of regions correlating MGMT promoter methylation and gene expression in glioblastomas. Neuro Oncol. Aug 2009;11(4):348–56. doi:10.1215/15228517-2009-001 19224763 PMC2743215

[31] Costello JF, Futscher BW, Kroes RA, Pieper RO. Methylation-related chromatin structure is associated with exclusion of transcription factors from and suppressed expression of the O-6-methylguanine DNA methyltransferase gene in human glioma cell lines. Mol Cell Biol. Oct 1994;14(10):6515–21. doi:10.1128/mcb.14.10.6515-6521.1994 7523853 PMC359181

[32] Pieper RO, Patel S, Ting SA, Futscher BW, Costello JF. Methylation of CpG island transcription factor binding sites is unnecessary for aberrant silencing of the human MGMT gene. J Biol Chem. Jun 7 1996;271(23):13916–24. doi:10.1074/jbc.271.23.13916 8662860

[33] Weller M, Felsberg J, Hartmann C, Molecular predictors of progression-free and overall survival in patients with newly diagnosed glioblastoma: a prospective translational study of the German Glioma Network. J Clin Oncol. Dec 1 2009;27(34):5743–50. doi:10.1200/JCO.2009.23.0805 19805672

[34] Perry JR, Laperriere N, O’Callaghan CJ, Short-Course Radiation plus Temozolomide in Elderly Patients with Glioblastoma. N Engl J Med. Mar 16 2017;376(11):1027–1037. doi:10.1056/NEJMoa1611977 28296618

[35] Stupp R, Hegi ME, Mason WP, Effects of radiotherapy with concomitant and adjuvant temozolomide versus radiotherapy alone on survival in glioblastoma in a randomised phase III study: 5-year analysis of the EORTC-NCIC trial. Lancet Oncol. May 2009;10(5):459–66. doi:10.1016/S1470-2045(09)70025-7 19269895

[36] Weller M, van den Bent M, Preusser M, EANO guidelines on the diagnosis and treatment of diffuse gliomas of adulthood. Nat Rev Clin Oncol. Mar 2021;18(3):170–186. doi:10.1038/s41571-020-00447-z 33293629 PMC7904519

[37] Wick W, Platten M, Meisner C, Temozolomide chemotherapy alone versus radiotherapy alone for malignant astrocytoma in the elderly: the NOA-08 randomised, phase 3 trial. Lancet Oncol. Jul 2012;13(7):707–15. doi:10.1016/S1470-2045(12)70164-X 22578793

[38] Mulholland S, Pearson DM, Hamoudi RA, MGMT CpG island is invariably methylated in adult astrocytic and oligodendroglial tumors with IDH1 or IDH2 mutations. Int J Cancer. Sep 1 2012;131(5):1104–13. doi:10.1002/ijc.26499 22020830

[39] Fleming JL, Pugh SL, Fisher BJ, Long-Term Report of a Comprehensive Molecular and Genomic Analysis in NRG Oncology/RTOG 0424: A Phase II Study of Radiation and Temozolomide in High-Risk Grade II Glioma. JCO Precis Oncol. 2021;5doi:10.1200/PO.21.00112 PMC8462570 34589661

[40] Dang L, White DW, Gross S, Cancer-associated IDH1 mutations produce 2-hydroxyglutarate. Nature. Dec 10 2009;462(7274):739–44. doi:10.1038/nature08617 19935646 PMC2818760

[41] Xu W, Yang H, Liu Y, Oncometabolite 2-hydroxyglutarate is a competitive inhibitor of alpha-ketoglutarate-dependent dioxygenases. Cancer Cell. Jan 18 2011;19(1):17–30. doi:10.1016/j.ccr.2010.12.014 21251613 PMC3229304

[42] Chowdhury R, Yeoh KK, Tian YM, The oncometabolite 2-hydroxyglutarate inhibits histone lysine demethylases. EMBO Rep. May 2011;12(5):463–9. doi:10.1038/embor.2011.43 21460794 PMC3090014

[43] Turcan S, Rohle D, Goenka A, IDH1 mutation is sufficient to establish the glioma hypermethylator phenotype. Nature. Feb 15 2012;483(7390):479–83. doi:10.1038/nature10866 22343889 PMC3351699

[44] Noushmehr H, Weisenberger DJ, Diefes K, Identification of a CpG island methylator phenotype that defines a distinct subgroup of glioma. Cancer Cell. May 18 2010;17(5):510–22. doi:10.1016/j.ccr.2010.03.017 20399149 PMC2872684

[45] Fisher BJ, Pugh SL, Macdonald DR, Phase 2 Study of a Temozolomide-Based Chemoradiation Therapy Regimen for High-Risk, Low-Grade Gliomas: Long-Term Results of Radiation Therapy Oncology Group 0424. Int J Radiat Oncol Biol Phys. Jul 15 2020;107(4):720–725. doi:10.1016/j.ijrobp.2020.03.027 32251755 PMC7456814

[46] van den Bent MJ, Tesileanu CMS, Wick W, Adjuvant and concurrent temozolomide for 1p/19q non-co-deleted anaplastic glioma (CATNON; EORTC study 26053–22054): second interim analysis of a randomised, open-label, phase 3 study. Lancet Oncol. Jun 2021;22(6):813–823. doi:10.1016/S1470-2045(21)00090-5 34000245 PMC8191233

[47] Jaeckle KA, Ballman KV, van den Bent M, CODEL: phase III study of RT, RT + TMZ, or TMZ for newly diagnosed 1p/19q codeleted oligodendroglioma. Analysis from the initial study design. Neuro Oncol. Mar 25 2021;23(3):457–467. doi:10.1093/neuonc/noaa168 32678879 PMC7992874

[48] Fink D, Aebi S, Howell SB. The role of DNA mismatch repair in drug resistance. Clin Cancer Res. Jan 1998;4(1):1–6. 9516945

[49] Hunter C, Smith R, Cahill DP, A hypermutation phenotype and somatic MSH6 mutations in recurrent human malignant gliomas after alkylator chemotherapy. Cancer Res. Apr 15 2006;66(8):3987–91. doi:10.1158/0008-5472.CAN-06-0127 16618716 PMC7212022

[50] Felsberg J, Thon N, Eigenbrod S, Promoter methylation and expression of MGMT and the DNA mismatch repair genes MLH1, MSH2, MSH6 and PMS2 in paired primary and recurrent glioblastomas. Int J Cancer. Aug 1 2011;129(3):659–70. doi:10.1002/ijc.26083 21425258

[51] Gestrich CK, Jajosky AN, Elliott R, Molecular Profiling of Pediatric and Adult Glioblastoma. Am J Clin Pathol. Mar 15 2021;155(4):606–614. doi:10.1093/ajcp/aqaa172 33210143

[52] Indraccolo S, Lombardi G, Fassan M, Genetic, Epigenetic, and Immunologic Profiling of MMR-Deficient Relapsed Glioblastoma. Clin Cancer Res. Mar 15 2019;25(6):1828–1837. doi:10.1158/1078-0432.CCR-18-1892 30514778

[53] Barthel FP, Johnson KC, Varn FS, Longitudinal molecular trajectories of diffuse glioma in adults. Nature. Dec 2019;576(7785):112–120. doi:10.1038/s41586-019-1775-1 31748746 PMC6897368

[54] Negm L, Chung J, Nobre L, The landscape of primary mismatch repair deficient gliomas in children, adolescents, and young adults: a multi-cohort study. Lancet Oncol. Jan 2025;26(1):123–135. doi:10.1016/S1470-2045(24)00640-5 39701117

[55] Das A, Sudhaman S, Morgenstern D, Genomic predictors of response to PD-1 inhibition in children with germline DNA replication repair deficiency. Nat Med. Jan 2022;28(1):125–135. doi:10.1038/s41591-021-01581-6 34992263 PMC8799468

[56] Cohen KJ, Pollack IF, Zhou T, Temozolomide in the treatment of high-grade gliomas in children: a report from the Children’s Oncology Group. Neuro Oncol. Mar 2011;13(3):317–23. doi:10.1093/neuonc/noq191 21339192 PMC3064602

[57] Jakacki RI, Cohen KJ, Buxton A, Phase 2 study of concurrent radiotherapy and temozolomide followed by temozolomide and lomustine in the treatment of children with high-grade glioma: a report of the Children’s Oncology Group ACNS0423 study. Neuro Oncol. Oct 2016;18(10):1442–50. doi:10.1093/neuonc/now038 27006176 PMC5035517

[58] Lulla RR, Buxton A, Krailo MD, Vorinostat, temozolomide or bevacizumab with irradiation and maintenance BEV/TMZ in pediatric high-grade glioma: A Children’s Oncology Group Study. Neurooncol Adv. Jan-Dec 2024;6(1):vdae035. doi:10.1093/noajnl/vdae035 38596718 PMC11003537

[59] Haase S, Banerjee K, Mujeeb AA, H3.3-G34 mutations impair DNA repair and promote cGAS/STING-mediated immune responses in pediatric high-grade glioma models. J Clin Invest. Nov 15 2022;132(22)doi:10.1172/JCI154229 PMC9663161 36125896

[60] Eckert A, Kloor M, Giersch A, Microsatellite instability in pediatric and adult high-grade gliomas. Brain Pathol. Apr 2007;17(2):146–50. doi:10.1111/j.1750-3639.2007.00049.x 17388945 PMC8095570

[61] Morano F, Raimondi A, Pagani F, Temozolomide Followed by Combination With Low-Dose Ipilimumab and Nivolumab in Patients With Microsatellite-Stable, O(6)-Methylguanine-DNA Methyltransferase-Silenced Metastatic Colorectal Cancer: The MAYA Trial. J Clin Oncol. May 10 2022;40(14):1562–1573. doi:10.1200/JCO.21.02583 35258987 PMC9084437

[62] Germano G, Lamba S, Rospo G, Inactivation of DNA repair triggers neoantigen generation and impairs tumour growth. Nature. Dec 7 2017;552(7683):116–120. doi:10.1038/nature24673 29186113

[63] Ercan AB, Aronson M, Fernandez NR, Clinical and biological landscape of constitutional mismatch-repair deficiency syndrome: an International Replication Repair Deficiency Consortium cohort study. Lancet Oncol. May 2024;25(5):668–682. doi:10.1016/S1470-2045(24)00026-3 38552658

[64] Crisafulli G, Sartore-Bianchi A, Lazzari L, Temozolomide Treatment Alters Mismatch Repair and Boosts Mutational Burden in Tumor and Blood of Colorectal Cancer Patients. Cancer Discov. Jul 6 2022;12(7):1656–1675. doi:10.1158/2159-8290.CD-21-1434 35522273 PMC9394384

[65] Chung J, Maruvka YE, Sudhaman S, DNA Polymerase and Mismatch Repair Exert Distinct Microsatellite Instability Signatures in Normal and Malignant Human Cells. Cancer Discov. May 2021;11(5):1176–1191. doi:10.1158/2159-8290.CD-20-0790 33355208 PMC8223607

[66] Westcott PMK, Muyas F, Hauck H, Mismatch repair deficiency is not sufficient to elicit tumor immunogenicity. Nat Genet. Oct 2023;55(10):1686–1695. doi:10.1038/s41588-023-01499-4 37709863 PMC10562252

[67] Bhatt D, Sundaram RK, Lopez KSL, Lee T, Gueble SE, Vasquez JC. Development of Syngeneic Murine Glioma Models with Somatic Mismatch Repair Deficiency to Study Therapeutic Responses to Alkylating Agents and Immunotherapy. Curr Protoc. Feb 2025;5(2):e70097. doi:10.1002/cpz1.70097 39995104 PMC12183790

[68] Wedge SR, Porteus JK, May BL, Newlands ES. Potentiation of temozolomide and BCNU cytotoxicity by O(6)-benzylguanine: a comparative study in vitro. Br J Cancer. Feb 1996;73(4):482–90. doi:10.1038/bjc.1996.85 8595163 PMC2074446

[69] Dolan ME, Moschel RC, Pegg AE. Depletion of mammalian O6-alkylguanine-DNA alkyltransferase activity by O6-benzylguanine provides a means to evaluate the role of this protein in protection against carcinogenic and therapeutic alkylating agents. Proc Natl Acad Sci U S A. Jul 1990;87(14):5368–72. doi:10.1073/pnas.87.14.5368 2164681 PMC54325

[70] Lee SM, Thatcher N, Crowther D, Margison GP. Inactivation of O6-alkylguanine-DNA alkyltransferase in human peripheral blood mononuclear cells by temozolomide. Br J Cancer. Mar 1994;69(3):452–6. doi:10.1038/bjc.1994.82 8123472 PMC1968858

[71] Quinn JA, Desjardins A, Weingart J, Phase I trial of temozolomide plus O6-benzylguanine for patients with recurrent or progressive malignant glioma. J Clin Oncol. Oct 1 2005;23(28):7178–87. doi:10.1200/JCO.2005.06.502 16192602

[72] Ranson M, Middleton MR, Bridgewater J, Lomeguatrib, a potent inhibitor of O6-alkylguanine-DNA-alkyltransferase: phase I safety, pharmacodynamic, and pharmacokinetic trial and evaluation in combination with temozolomide in patients with advanced solid tumors. Clin Cancer Res. Mar 1 2006;12(5):1577–84. doi:10.1158/1078-0432.CCR-05-2198 16533784

[73] Quinn JA, Jiang SX, Reardon DA, Phase II trial of temozolomide plus o6-benzylguanine in adults with recurrent, temozolomide-resistant malignant glioma. J Clin Oncol. Mar 10 2009;27(8):1262–7. doi:10.1200/JCO.2008.18.8417 19204199 PMC2667825

[74] Ranson M, Hersey P, Thompson D, Randomized trial of the combination of lomeguatrib and temozolomide compared with temozolomide alone in chemotherapy naive patients with metastatic cutaneous melanoma. J Clin Oncol. Jun 20 2007;25(18):2540–5. doi:10.1200/JCO.2007.10.8217 17577032

[75] Khan OA, Ranson M, Michael M, A phase II trial of lomeguatrib and temozolomide in metastatic colorectal cancer. Br J Cancer. May 20 2008;98(10):1614–8. doi:10.1038/sj.bjc.6604366 18475294 PMC2391129

[76] Ramalho MJ, Loureiro JA, Coelho MAN, Pereira MC. Factorial Design as a Tool for the Optimization of PLGA Nanoparticles for the Co-Delivery of Temozolomide and O6-Benzylguanine. Pharmaceutics. Aug 10 2019;11(8) doi:10.3390/pharmaceutics11080401 PMC6722980 31405159

[77] Hegde MM, Palkar P, Mutalik SP, Mutalik S, Goda JS, Rao BSS. Enhancing glioblastoma cytotoxicity through encapsulating O6-benzylguanine and temozolomide in PEGylated liposomal nanocarrier: an in vitro study. 3 Biotech. Nov 2024;14(11):275. doi:10.1007/s13205-024-04123-2 PMC11499494 39450422

[78] Liang S, Xu H, Ye BC. Membrane-Decorated Exosomes for Combination Drug Delivery and Improved Glioma Therapy. Langmuir. Jan 11 2022;38(1):299–308. doi:10.1021/acs.langmuir.1c02500 34936368

[79] Zhang J, Stevens MF, Hummersone M, Madhusudan S, Laughton CA, Bradshaw TD. Certain imidazotetrazines escape O6-methylguanine-DNA methyltransferase and mismatch repair. Oncology. 2011;80(3–4):195–207. doi:10.1159/000327837 21720182

[80] Zhang J, Hummersone M, Matthews CS, Stevens MF, Bradshaw TD. N3-substituted temozolomide analogs overcome methylguanine-DNA methyltransferase and mismatch repair precipitating apoptotic and autophagic cancer cell death. Oncology. 2015;88(1):28–48. doi:10.1159/000366131 25277441

[81] Cousin D, Hummersone MG, Bradshaw TD, Synthesis and growth-inhibitory activities of imidazo[5,1-d]-1,2,3,5-tetrazine-8-carboxamides related to the anti-tumour drug temozolomide, with appended silicon, benzyl and heteromethyl groups at the 3-position. Medchemcomm. Mar 1 2018;9(3):545–553. doi:10.1039/c7md00554g 30108945 PMC6072467

[82] Summers HS, Lewis W, Williams HEL, Bradshaw TD, Moody CJ, Stevens MFG. Discovery of new imidazotetrazinones with potential to overcome tumor resistance. Eur J Med Chem. Sep 5 2023;257:115507. doi:10.1016/j.ejmech.2023.115507 37262998

[83] Svec RL, McKee SA, Berry MR, Kelly AM, Fan TM, Hergenrother PJ. Novel Imidazotetrazine Evades Known Resistance Mechanisms and Is Effective against Temozolomide-Resistant Brain Cancer in Cell Culture. ACS Chem Biol. Feb 18 2022;17(2):299–313. doi:10.1021/acschembio.2c00022 35119837 PMC9624299

[84] Yang Z, Wei D, Dai X, C8-Substituted Imidazotetrazine Analogs Overcome Temozolomide Resistance by Inducing DNA Adducts and DNA Damage. Front Oncol. 2019;9:485. doi:10.3389/fonc.2019.00485 31263673 PMC6584802

[85] Svec RL, Furiassi L, Skibinski CG, Fan TM, Riggins GJ, Hergenrother PJ. Tunable Stability of Imidazotetrazines Leads to a Potent Compound for Glioblastoma. ACS Chem Biol. Nov 16 2018;13(11):3206–3216. doi:10.1021/acschembio.8b00864 30296373 PMC6243397

[86] Lin K, Gueble SE, Sundaram RK, Huseman ED, Bindra RS, Herzon SB. Mechanism-based design of agents that selectively target drug-resistant glioma. Science. Jul 29 2022;377(6605):502–511. doi:10.1126/science.abn7570 35901163 PMC9502022

[87] Tong WP, Kirk MC, Ludlum DB. Mechanism of action of the nitrosoureas--V. Formation of O6-(2-fluoroethyl)guanine and its probable role in the crosslinking of deoxyribonucleic acid. Biochem Pharmacol. Jul 1 1983;32(13):2011–5. doi:10.1016/0006-2952(83)90420-3 6870930

[88] Pegg AE, Scicchitano D, Dolan ME. Comparison of the rates of repair of O6-alkylguanines in DNA by rat liver and bacterial O6-alkylguanine-DNA alkyltransferase. Cancer Res. Sep 1984;44(9):3806–11. 6378375

[89] Parker S, Kirk MC, Ludlum DB. Synthesis and characterization of O6-(2-chloroethyl)guanine: a putative intermediate in the cytotoxic reaction of chloroethylnitrosoureas with DNA. Biochem Biophys Res Commun. Nov 13 1987;148(3):1124–8. doi:10.1016/s0006-291x(87)80249-8 3689390

[90] Huseman ED, Lo A, Fedorova O, Mechanism of Action of KL-50, a Candidate Imidazotetrazine for the Treatment of Drug-Resistant Brain Cancers. J Am Chem Soc. Jul 10 2024;146(27):18241–18252. doi:10.1021/jacs.3c06483 38815248 PMC11409917

[91] Wang P, Wu J, Ma S, Oncometabolite D-2-Hydroxyglutarate Inhibits ALKBH DNA Repair Enzymes and Sensitizes IDH Mutant Cells to Alkylating Agents. Cell Rep. Dec 22 2015;13(11):2353–2361. doi:10.1016/j.celrep.2015.11.029 26686626 PMC4694633

[92] Sulkowski PL, Corso CD, Robinson ND, 2-Hydroxyglutarate produced by neomorphic IDH mutations suppresses homologous recombination and induces PARP inhibitor sensitivity. Sci Transl Med. Feb 1 2017;9(375)doi:10.1126/scitranslmed.aal2463 PMC5435119 28148839

[93] Sulkowski PL, Oeck S, Dow J, Oncometabolites suppress DNA repair by disrupting local chromatin signalling. Nature. Jun 2020;582(7813):586–591. doi:10.1038/s41586-020-2363-0 32494005 PMC7319896

[94] Schvartzman JM, Forsyth G, Walch H, Oncogenic IDH mutations increase heterochromatin-related replication stress without impacting homologous recombination. Mol Cell. Jul 6 2023;83(13):2347–2356 e8. doi:10.1016/j.molcel.2023.05.026 37311462 PMC10845120

[95] Herrlinger U, Tzaridis T, Mack F, Lomustine-temozolomide combination therapy versus standard temozolomide therapy in patients with newly diagnosed glioblastoma with methylated MGMT promoter (CeTeG/NOA-09): a randomised, open-label, phase 3 trial. Lancet. Feb 16 2019;393(10172):678–688. doi:10.1016/S0140-6736(18)31791-4 30782343

[96] A Phase III Trial of Lomustine-Temozolomide Combination Therapy Versus Standard Temozolomide in Patients With Methylated MGMT Promoter Glioblastoma. National Library of Medicine (US). https://clinicaltrials.gov/study/NCT05095376

